# Supplementary material for: Combination of Solid State and Submerged Fermentation Strategies to Produce a New Jellyfish-Based Food
Source: Foods. 2022 Dec 8;11(24):3974. doi: 10.3390/foods11243974 (PMC9778331; doi:10.3390/foods11243974)
Supplement: Supplementary file 1 [file foods-11-03974-s001.zip › foods-2057783-supplementary.pdf]

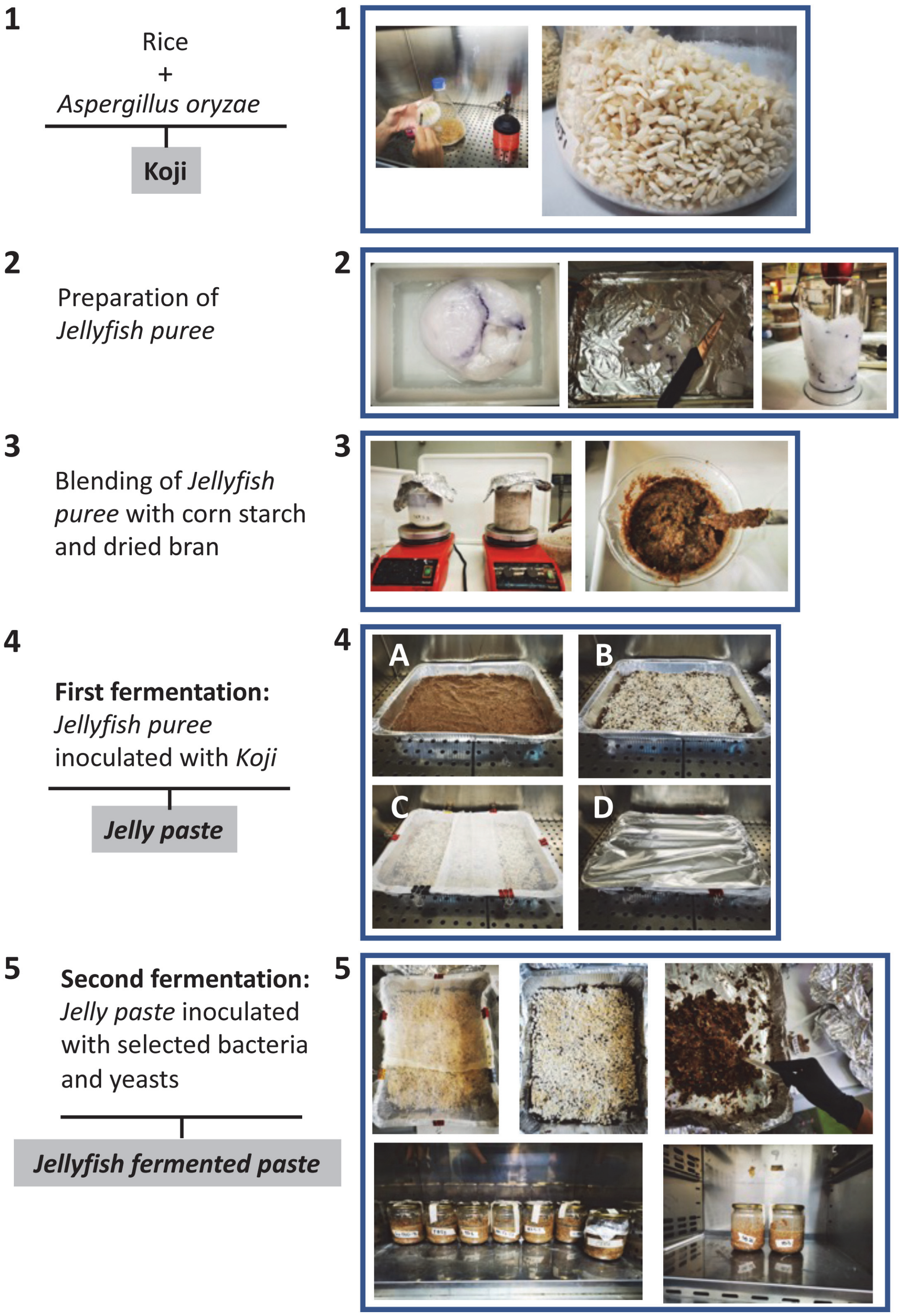

Figure S1. Procedure for producing fermented Jellyfish paste described per images. 1: Inoculation of the fungal starter *Aspergillus oryzae* in *Carnaroli* rice to produce Koji. 2: Preparation of a Jellyfish puree from *Rhizostoma pulmo* raw material. 3: Assembly of Jellyfish paste by mixing jellyfish puree, corn starch and wheat bran as ingredients. 4: Inoculation with Koji from step 1 of the jellyfish puree and solid-state fermentation (first fermentation) for two days in the dark to obtain the Jelly paste. 5: Sterilization of Jelly paste, inoculation of the selected bacteria and yeasts strains for the submerged fermentation (second fermentation) for 10 days to obtain fermented Jellyfish pastes.
